# Robust and fast SSFP for the evaluation of LV function at 3T

**DOI:** 10.1186/1532-429X-15-S1-O52

**Published:** 2013-01-30

**Authors:** Yin Wu, Fan Yang, Yiu-Cho Chung

**Affiliations:** 1 Paul C. Lauterbur Research Centre for Biomedical Imaging, Shenzhen Key Laboratory for MRI, Shenzhen Institutes of Advanced Technology, Shenzhen, China

## Background

3T MRI is advantageous to CMR as it offers higher SNR and better T1 contrast. Its slow clinical adoption is partly due to the lack of robustness in SSFP cine imaging [1]. Banding artifact in SSFP caused by main field inhomogeneity is common at 3T. Wideband SSFP [2] or prescans (shimming, frequency scout, etc.) are needed to avoid/reduce the artifact, increasing patient scan time. We propose here to use shorter TR for SSFP through slightly reduced spatial resolution to improve the sequence's robustness and speed for cine imaging. Its ability for LV function assessment was evaluated against the standard SSFP acquisition protocol.

## Methods

### Protocol

A 600 µs (instead of 1000 µs) RF pulse and 192 (instead of 256) readout point in SSFP shortened the TR/TE from 3.4/1.5 ms to 2.7/1.2 ms. At 40 ms temporal resolution and 85% phase resolution, the segments/heart beat increased from 12 to 15, reducing patient's breath-hold time.

### Imaging

The IRB approved study scanned 8 healthy volunteers (age 26±2) with a 3T MR scanner (TIM TRIO, Siemens, Germany). Cines of 10 short-axis slices covering the whole heart were acquired with retrogated breath-held SSFP (8mm thick, 2mm gap, FOV=340×287mm^2, iPAT=2, maximum bandwidth). The short TR sequence was used first. Frequency scout was then performed. After that, cine acquisition for the whole heart was repeated using the standard protocol. LV myocardial mass, end-diastolic volume (EDV), end-systole volume (ESV) and ejection fraction (EF) were analyzed by an experienced observer using QMass MR (Medis, Netherlands) (papillary muscles included). Correlation analysis was performed between paired measurements. Two-tailed paired t-test was conducted to assess the statistical differences with p<0.05 regarded as significant.

## Results

The scan time for 1 slice of cine was about 6 and 9 heart beats for the protocols with short and standard TR respectively. Banding artifact was common in cine images obtained with the standard TR protocol, while images from the protocol using short TR scan were impervious to the artifact. Analysis results were shown in Table 1. Myocardial mass, EDV and ESV from the 2 protocols were very close. The short TR protocol yielded a 1% reduction in EF compared to the standard one.

**Table 1:** LV function measured from the two SSFP protocols.

	Short TR	Standard TR	p-value	r
Myocardial mass (g)	101.2±12.2	100.2±12.4	ns	0.99
EDV (ml)	117.4±20.4	118.0±19.9	ns	0.99
ESV (ml)	37.8±8.1	36.8±7.9	ns	0.99
EF (%)	67.9±3.2	68.9±3.5	<0.05	0.99

Note: ns - not significant.

## Conclusions

The short TR SSFP cine is more robust to field inhomogeneity at 3T. It eliminates frequency scout and increases patient throughput. Initial results showed that short TR cines gave comparable myocardial mass, EDV and ESV. The measured EF was 1% lower than the standard protocol, and may be caused by the reduced image spatial resolution. Our preliminary study suggested that by slightly trading spatial resolution for shorter TR in SSFP, cine imaging at 3T can be more robust and efficient. The approach may greatly facilitate patient throughput in 3T CMR. Evaluation on more volunteers and patients will be done to fully validate the method.

## Funding

None.

**Figure 1:**
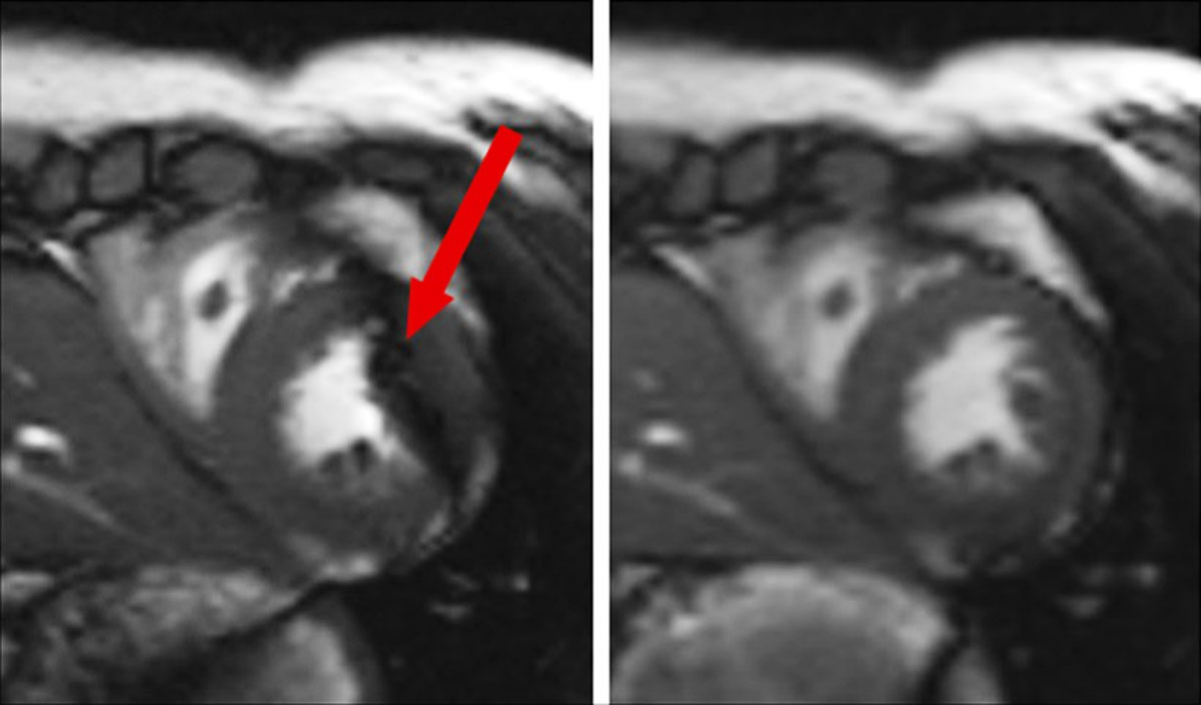
A typical cine image set obtained from SSFP with standard TR (left) and short TR (right). Banding artifact easily appeared in images using the standard TR protocol, while the scan with short TR was impervious to such artifact.
